# Biostimulants alleviate water deficit stress and enhance essential oil productivity: a case study with savory

**DOI:** 10.1038/s41598-022-27338-w

**Published:** 2023-01-13

**Authors:** Esmaeil Rezaei-Chiyaneh, Hassan Mahdavikia, Hadi Alipour, Aria Dolatabadian, Martin Leonardo Battaglia, Sagar Maitra, Matthew Tom Harrison

**Affiliations:** 1 Department of Plant Production and Genetics, Faculty of Agriculture, Urmia University, Urmia, Iran; 2 Department of Medicinal Plants, Shahid Bakeri Higher Education Center of Miandoab, Urmia University, Urmia, Iran; 3 School of Biological Sciences, The University of Western Australia, 35 Stirling Highway, Crawley, WA 6009 Australia; 4 Department of Animal Science, Cornell University, Ithaca, NY 14853 USA; 5 Centurion University of Technology and Management, Sitapur, Odisha 761211 India; 6 Tasmanian Institute of Agriculture, University of Tasmania, Newnham, Launceston, 7248 Australia

**Keywords:** Biochemistry, Physiology

## Abstract

Water deficit stress exposure frequently constrains plant and agri-food production globally. Biostimulants (BSs) can be considered a new tool in mitigating water deficit stress. This study aimed to understand how BSs influence water deficit stress perceived by savory plants (*Satureja hortensis* L.), an important herb used for nutritional and herbal purposes in the Middle East. Three BS treatments, including bio-fertilizers, humic acid and foliar application of amino acid (AA), were implemented. Each treatment was applied to savory plants using three irrigation regimes (low, moderate and severe water deficit stress FC100, FC75 and FC50, respectively). Foliar application of AA increased dry matter yield, essential oil (EO) content and EO yield by 22%, 31% and 57%, respectively. The greatest EO yields resulted from the moderate (FC75) and severe water deficit stress (FC50) treatments treated with AA. Primary EO constituents included carvacrol (39–43%), gamma-terpinene (27–37%), alpha-terpinene (4–7%) and *p*-cymene (2–5%). Foliar application of AA enhanced carvacrol, gamma-terpinene, alpha-terpinene and *p*-cymene content by 6%, 19%, 46% and 18%, respectively. Physiological characteristics were increased with increasing water shortage and application of AA. Moreover, the maximum activities of superoxide dismutase (3.17 unit mg^−1^ min^−1^), peroxidase (2.60 unit mg^−1^ min^−1^) and catalase (3.08 unit mg^−1^ min^−1^) were obtained from plants subjected to severe water deficit stress (FC50) and treated with AA. We conclude that foliar application of AA under water deficit stress conditions would improve EO quantity and quality in savory.

## Introduction

Arable environments across much of Iran often subject plants to water deficit stress, with the country having an average annual rainfall ranging between 224 and 275 mm^1,2^. Exposure to drought (here defined as lack of plant available water for a sustained period) inhibits plant growth and development by changing plant physiological and biochemical activities, resulting in productivity and quality losses^3–8^. Physiologically, drought can reduce photosynthetic rates, enhance phenology, truncate root growth and enhance senescence^9–11^. Exposure to enduring reactive oxygen species (ROS) such as superoxide anion, hydroxyl radical, hydrogen peroxide and singlet oxygen may cause oxidative damage and cell death^12,13^. Degrading chlorophyll due to excessive ROS also decreases the photosynthetic rate and plant productivity^14^. To deal with stress exposure, plants have evolved various physiological and biochemical mechanisms to decrease the harmful impacts of ROS. For example, producing osmolytes or osmoprotectant compounds such as proline, glycine betaine and phenolic compounds is a well-regulated biochemical mechanism for enhancing plant tolerance against drought stress conditions^15^.

Bio-stimulants (BSs), including natural substances such as proline, glycine betaine and glutamic acid, could potentially be used to alleviate water stress effects. However, their effects on plant productivity and food quality are poorly studied under water deficit. Some studies have shown that using BS decreases the negative impacts of drought stress by improving nutrient uptake efficiency and tolerance to stress through enhanced osmotic adjustment^16^. In contrast, other studies have revealed more positive impacts of BS on mitigating drought stress than synthetic chemicals^17^. Recent research has shown that BS applied as organic and bio-fertilizers may enhance the nutrient use efficiency in plants subjected to drought stress compared to chemical fertilizer application^18^. Furthermore, BSs regulate the nutrient acquisition and secretion of phytohormones such as auxin, cytokinin, and gibberellin, promote nitrogen-fixing bacteria and enhance potassium and phosphorus solubilization, potentially improving soil structure and aggregation and increase plant productivity^19^.

The global community's interest in using medicinal and aromatic plants (MAP) as natural alternatives to chemical drugs against medical disorders is growing, primarily because such plants contain essential oils (EOs), vitamins and minerals. Essential oils are natural volatile aroma compounds synthesized by aromatic plants and have been used in the medicine, cosmetics and food industries^20^. One such MAP is summer savory (*Satureja hortensis* L.), an annual species belonging to the Lamiaceae family with a pleasant aroma and high EO content. Previous work has shown that the main EO constituents of savory include carvacrol, *α*-thujune, *p*-cymene, *β*-myrcene, α-pinene, *β*-terpinene, thymol, linalool, and *β*-caryophyllene^21,22^. Savory has also been used in traditional medicine as a painkiller to treat ailments related to coughing, indigestion, diarrhea, nausea and loss of appetite^22^. Also, the relatively high vitamin C and vitamin A content of savory may contribute to long-term health by boosting the natural state of the human immune system.

For many arid and semi-arid regions in which MAP are traditionally grown, farmer financial income may be threatened by potential low yields and product quality losses. So multiple avenues have been proposed for sustainably raising farmer income under drought^23,24^. Across plant species, management options and geographical location, drought stress has been reported to have substantial effects on biomass production^25,26^. We propose using natural compounds as bio-fertilizers would reduce production costs and potentially maintain plant production under drought stress. Hence, the present study aimed to investigate the effects of fertilizers (organic and bio-fertilizer) and BS compounds on the productivity and phytochemical properties of savory under drought-stress conditions.

## Materials and methods

### Experimental area

Two field experiments were conducted in 2020 and 2021 at a farm in the Higher Education Center of Shahid Bakeri Miandoab, Iran. Weather (mean temperature and annual precipitation) and soil physicochemical characteristics (taken at the 0–30 cm depth) of the experimental area are shown in Tables 1 and 2, respectively.

**Table 1:** Meteorological data from March to July in 2020 and 2021 in the study site at Miandoab, Iran.

Year	March	April	May	June	July	August
**Monthly average temperature (°C)**						
2020	10.1	16.1	21.8	24.8	25	21.9
2021	12.6	19.1	22.6	26.3	25.6	22.6
**Monthly average precipitation (mm)**						
2020	0.82	0.92	0.01	0.03	0	0.11
2021	0.27	0.97	0.05	0	0.14	0
**Monthly average relative humidity (%)**						
2020	65	55	37	43	48	52
2021	48	47	37	39	45	42

**Table 2:** Soil chemical properties.

Texture	pH	EC (dS m^−1^)	Organic matter (%)	Total N (%)	Phosphorus (%)	Potassium (%)
Silty	7.92	0.69	1.08	0.09	11.21	212.31

### Plots preparation, treatments and cultivation

The experiments were established in a randomized complete block design (RCBD) with a split-plot arrangement and three replicates. Each subplot was composed of 10 rows of savory with an inter-row spacing of 40 cm. There was a 25 cm distance between plants within the rows. The subplots were 2.5 × 4 m. Plots and blocks were separated by a buffer space of 2 m and 3 m, respectively. Main plots were allocated to different irrigation regimes (3 levels), including full irrigation (FC100), moderate (FC75) and severe water stress (FC50), while sub-plots were assigned to different fertilizer sources (4 levels), including no fertilization (control), humic acid (HA), bio-fertilizer (B) bacteria [mixture of phosphate-solubilizing bacteria (PSB) *Pantoea agglomerans* + *Pseudomonas putida*, K-solubilizing bacteria (KSB) *Pseudomonas koreensis* + *Pseudomonas vancouverensis,* and N-fixing bacteria (NFB) *Azotobacter vinelandii*] and foliar application of amino acid (AA).

The savory (Miandoab local landraces) seeds were obtained from Urmia University, Iran. For bio-fertilizer treatments, savory seeds were soaked in biofertilizer agents (10^8^ active bacteria per g) and dried in the shade for one h before seeding. Seeds were manually sown on May 4, 2020, and May 6, 2021. There was a 25 cm distance between plants within the rows. Weeds were regularly removed by hand when needed.

Each experimental unit utilised a total application rate of 200 mg of the HA powder for the HA treatment. One-third of this amount was dissolved in water and applied at seed sowing, while the remaining two-thirds were added to the water and used at the stem elongation and later at the flowering stage. The HA treatment was added in the respective experimental units with the first irrigation (5–10 L ha^−1^; after seed sowing and before flowering). The HA treatment (Humax® 95–WSG) contained 80% humic acid, 15% folic acid, and 12% potassium. Foliar application of the AA treatment (43.7% total protein, 50% amino acids, and 20% total N) was performed twice in each growing season, once at the beginning of the shooting stage and once four weeks later at the concentration of 2 L per 1000 L of water. Spraying was done in the afternoon near sunset. Control plants were sprayed using distilled water.

The plant material and seeds were obtained under the supervision and permission of Urmia University and according to national guidelines, and all authors comply with all the local and national guidelines.

### Measurements

#### Agronomic traits

Ten plants were randomly selected from each plot at the full flowering stage to determine agronomic traits, including plant height, canopy diameter, and lateral branch number. For dry matter determination, a 1.6 m^2^ area was harvested at each plot on August 10, 2020, and August 14, 2021.

#### Essential oil extraction and analysis

The savory EO was extracted using the water distillation method by a Clevenger apparatus. For this, 40 g of dried plant material (leaves + flowers) was added to the balloon of the essential oil extraction machine and 500 ml of distilled water was added. The essential oil extraction operation was carried out for three hours. The extracted EO was dried with anhydrous sodium sulfate and kept at 4 °C for further chemical analysis. EO yield (kg ha^−1^) was calculated by multiplying the dry matter yield by its EO percentage. Oil constituents were analyzed using a GC–MS (Agilent 7890/5975A), as suggested by Namazi et al.^27^.

#### Relative water content

At the flower initiation stage, the leaves' relative water content (RWC) was determined according to Levitt^28^, as follows:

$$\text{RWC}\; (\%)=\frac{\mathrm{Fw}-\mathrm{Dw}}{\mathrm{Tw}-\mathrm{Dw}}\times 100$$

where F_w_, T_w,_ and D_w_ represent the fresh, turgid (by floating the fresh leaves in double distilled water at 4 °C for 5 h) and dry weight of leaves (by drying the leaves in an oven at 70 °C for 48 h), respectively.

#### Physiological characteristics

In the flowering stage, 0.5 g fresh savory leaves were homogenized in 10 mL of 80% acetone and centrifuged at 12,000 rpm for 15 min. After that, the absorbance was read at 663 nm, 645 nm and 470 nm by a UV spectrophotometer (UV-1800, Shimadzu, Tokyo, Japan). The content of chlorophyll a, b and carotenoids was calculated based on the following equations^29^:

$$\begin{aligned} & {\text{Ch1}}a\, = \,\left( {{12}.{\text{25A}}_{{{663}.{2}}} } \right)\, - \,\left( {{2}.{\text{79A}}_{{{646}.{8}}} } \right) \\ & {\text{Ch1}}b\, = \,\left( {{21}.{\text{5A}}_{{{646}.{8}}} } \right)\, - \,\left( {{5}.{\text{1A}}_{{{663}.{2}}} } \right) \\ & {\text{Car}} = \frac{{\left[ {1000{\text{A}}470 - 1.82{\text{Ca}} - 85.02{\text{Cb}}} \right]}}{198} \\ \end{aligned}$$

In these equations, Chl *a*, *b* and Car represent chlorophyll *a*, *b* and carotenoid concentrations, respectively. Parameters A646.8, A663.2 and A470 represent the absorbance measured at 646.8, 663.2 and 470 nm using a spectrophotometer.

Proline concentration was determined following the method described by Bates et al.^30^. First, 0.5 g of fresh leaves were homogenized with 10 mL of 3% sulfosalicylic acid and centrifuged at 12,000*g* for 10 min at 4 °C. Then, 2 mL of each sample was boiled with 2 mL of acid-ninhydrin + 2 mL of glacial acetic acid for 40 min. The reaction mixture was extracted with 4 mL of toluene and mixed with a vortex for 20 s. Finally, the absorbance was measured spectrophotometrically at 520 nm compared to the clean toluene. The soluble sugar concentration (SSC) was determined using the phenol and sulfuric acid method. Briefly, 0.5 g of savory fresh leaves were homogenized with ethanol and mixed with 98% sulfuric acid and 5% phenol and absorbance were determined with a spectrophotometer at 485 nm^31^.

First, to assess the antioxidant enzyme activity across different irrigation levels and fertilizer sources, 0.1 g of savory leaves (frozen in liquid nitrogen) were homogenized in 2 mL sodium phosphate buffer and centrifuged at 15,000 rpm at 4 °C for 20 min. Then, the supernatant was used to determine antioxidant enzyme activity. The superoxide dismutase (SOD), peroxidase (POX) and catalase (CAT) activities were measured according to the procedures reported by Dhindsa et al.^32^, Kar and Feierabend^33^ and Aebi^34^, respectively. All enzyme activity was reported in units of mg g^−1^ min^−1^.

### Antioxidant activity

The 2,2-Diphenyl-1-Picrylhydrazyl (DPPH) assay was utilized to determine the plant's antioxidant activity. For this purpose, 3.8 mg of DPPH was dissolved in 25 mL MeOH. Next, 100 µL of the mixture was added into individual wells of 96-well plates and mixed with 85 µL MeOH and 15 µL of savory sample extract. Finally, the microplates were incubated in the dark and at room temperature for 30 min, and the absorbance was read at 515 nm^35^.

### Statistical analysis

A combined analysis of variance (ANOVA) and the comparison of the means with Duncan’s multiple range test at a 5% probability level were performed using the SAS software version 9.4 (SAS Institute Inc., Cary, NC, USA). The irrigation levels and fertilizer sources were considered fixed effects, while block, year, and interactions were considered random. Correlation coefficients among all variables under analysis were also calculated using the SAS software version 9.4 (SAS Institute Inc., Cary, NC, USA). The heatmap clusters were created in Ward’s method using heatmap.2 functions in gplots R-package.

## Results

The analysis of variance in Tables 3 and 4 show that the main effects of “Irrigation” (I) and “Fertilizer” (F) were significant for all the agronomical (Table 3) and physiological (Table 4) traits under analysis. However, the interaction between both factors was also significant in all cases, so this is the effect that was analyzed for all traits under study.

**Table 3:** Effects of irrigation regimes and biofertilizer application on agronomical characteristics of savory.

S.O.V	d.f	Mean squares (MS)					
		Plant height	Lateral branch	Dry matter yield	Canopy diameter	Essential oil content	Essential oil yield
Year (Y)	1	535.23**	8.96^ns^	1,733,049.05**	318.61**	0.7640**	568.24*
R(Y)	4	7.23	1.18	25,104.68	8.09	0.0185	29.27
Irrigation (I)	2	1109.30**	132.27**	17,027,238.34**	1140.64**	0.6940**	96.39**
Y × I	2	117.32^ns^	9.93*	378,154.03**	59.11**	0.1811**	373.53**
R(Y × I)	8	28.71	1.49	21,609.89	3.94	0.0037	9.91
Fertilizer (F)	3	192.60**	95.78**	2,132,742.23**	155.26**	0.1836**	855.34**
Y × F	3	3.40^ns^	1.48^ns^	6650.42^ns^	3.89**	0.0646**	96.30**
I × F	6	16.50**	5.88**	119,219.00**	17.86**	0.0448**	108.97**
Y × I × F	6	3.66^ns^	0.50^ns^	11,918.81^ns^	2.80**	0.0210**	27.73**
Error	36	2.10	0.68	6671.31	0.45	0.0017	4.12

*S.O.V* source of variation, *R* replication, *d.f* degree of freedom.

ns, * and **: non-significant, significant at 5% and 1% probability level, respectively.

**Table 4:** Effects of irrigation regimes and biofertilizer application on physiological characteristics of savory.

S.O.V	d.f	Mean squares (MS)									
		Chlorophyll *a*	Chlorophyll *b*	Carotenoids content	Relative water content	Proline content	Soluble sugars content	CAT	SOD	POX	Antioxidant activity
Year (Y)	1	0.5540*	0.8391**	0.2230*	3434.49**	0.8712**	0.0756^ns^	0.0030^ns^	0.0181^ns^	0.0691^ns^	0.0141**
R(Y)	4	0.0374	0.0051	0.0246	15.86	0.0384	0.0265	0.1019	0.0056	0.0924	0.1335
Irrigation (I)	2	3.1542**	0.6742**	1.2399**	3121.27**	7.7936**	29.8193**	8.0117**	14.0658**	2.5533**	176.9434**
Y × I	2	0.0149^ns^	0.0130^ns^	0.0141^ns^	72.27*	0.0215^ns^	0.0027^ns^	0.0335^ns^	0.0053^ns^	0.0081^ns^	0.1263^ns^
R(Y × I)	8	0.0174	0.0046	0.0150	15.66	0.0074	0.0167	0.0397	0.0179	0.0348	0.4542
Fertilizer (F)	3	0.8163**	0.2404**	0.5991**	309.43**	1.3938**	1.8632**	1.4363**	1.9930**	0.8385**	37.2307**
Y × F	3	0.0178^ns^	0.0014^ns^	0.0041^ns^	13.76**	0.0065^ns^	0.0070^ns^	0.0044^ns^	0.0201^ns^	0.0009^ns^	0.2757^ns^
I × F	6	0.0990**	0.0183**	0.1090**	46.06**	0.2865**	0.4540**	0.3189**	0.4401**	0.2576**	3.7983**
Y × I × F	6	0.0096^ns^	0.0020^ns^	0.0015^ns^	1.74^ns^	0.0069^ns^	0.0106^ns^	0.0045^ns^	0.0081^ns^	0.0060^ns^	0.2194^ns^
Error	36	0.0095	0.0031	0.0044	2.42	0.0142	0.0231	0.0101	0.0116	0.0083	0.1756

*S.O.V* source of variation, *R* replication, *d.f* degree of freedom.

ns, * and **: non-significant, significant at 5% and 1% probability level, respectively.

### Plant height

**Figure 1:**
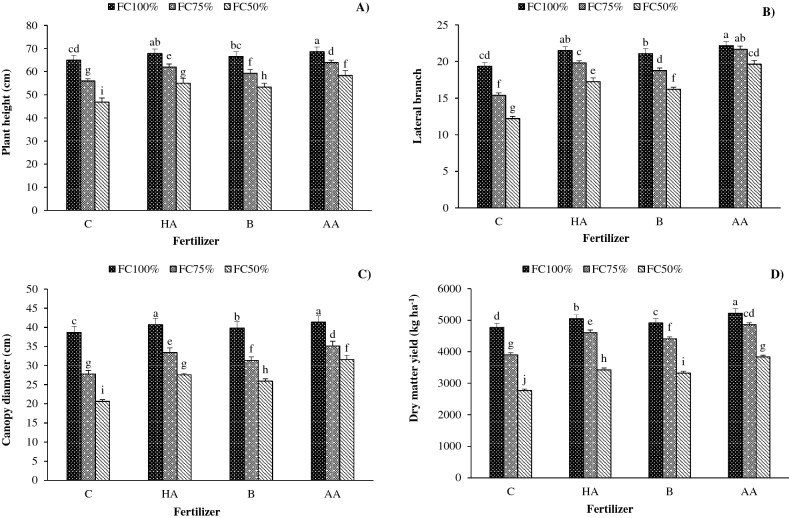
Interaction effects of irrigation regime and biofertilizer application on (**A**) plant height, (**B**) lateral branch, (**C**) canopy diameter, and (**D**) dry matter yield in savory. *C* control, *HA* humic acid, *B* bacterial, *AA* amino acid. Different lower-case letters above bars indicate significant differences at *P* < 0.05.

The maximum plant height (68.7 cm) was obtained from FC100 treatment fertilized with AA followed by HA application (67.8 cm). However, the minimum plant height (46.8 cm) was achieved from FC50 treatment without fertilization (control). Compared with FC100, plant height decreased in FC75 and FC50 by 10% and 20%, respectively. In addition, plant height was enhanced by 10%, 7% and 14% due to HA, B and AA application (Fig. 1A).

### Lateral branch number

The maximum lateral branch number (22.2) was obtained from FC100 treatment fertilized with AA. In contrast, the minimum lateral branch number (12.2) was related to FC50 treatment without fertilization (control). Lateral branch numbers decreased in FC75 and FC50 by 10% and 22%, respectively. Moreover, lateral branch numbers increased by 25%, 19% and 35% due to HA, B and AA applications (Fig. 1B).

### Canopy diameter

The highest canopy diameter (41.4 cm) was obtained from FC100 treatment fertilized with AA, followed by HA application (40.7 cm). However, the lowest canopy diameter (20.6 cm) was achieved from FC50 without fertilization (control). Compared with FC100, the canopy diameter decreased by 20% and 34% in FC75 and FC50 water stress. In addition, fertilization with HA, B and AA increased canopy diameter by 17%, 11% and 24%, respectively (Fig. 1C).

### Dry matter yield (DMY)

The maximum (5225 kg ha^−1^) and minimum (2767 kg ha^−1^) DMY was achieved from FC100 treatment fertilized with AA and FC100 without fertilization. The DMY decreased by increasing water deficit stress severity. Compared to the FC100 treatment, DMY decreased in FC75 and FC50 treatments by 11% and 33%, respectively. In addition, fertilization with HA, B and AA increased canopy diameter by 14%, 11% and 22%, respectively (Fig. 1D).

### Essential oil (EO) content and yield

**Figure 2:**
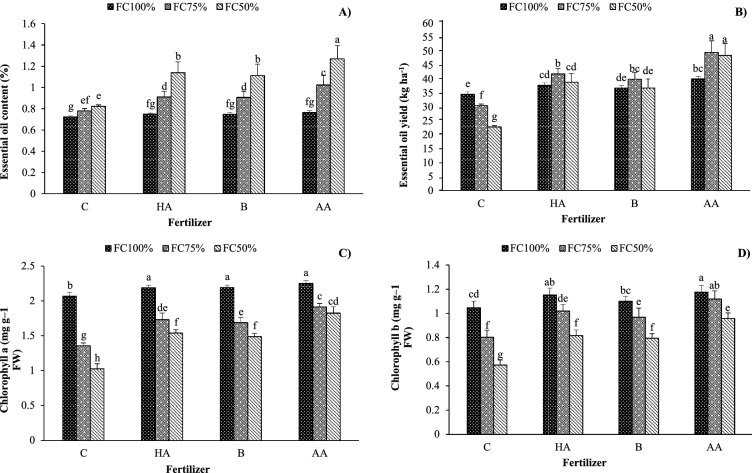
Interaction effects of irrigation regime and biofertilizer application on (**A**) essential oil content, (**B**) **e**ssential oil yield, (**C**) chlorophyll *a*, and (**D**) chlorophyll *b* in savory. *C* control, *HA* humic acid, *B* bacterial, *AA* amino acid. Different lower-case letters above bars indicate significant differences at *P* < 0.05.

The EO content extracted from the aerial parts was the highest (1.27%) in the FC50 treatment treated with AA. However, the lowest EO content (0.72%) was recorded in no-stress conditions (FC100) without fertilization. Across irrigation regimes, EO content increased by 21% and 45% in moderate (FC75) and severe (FC50) water deficit stress treatments. Also, the EO content increased by 19%, 18% and 31% due to HA, B and AA applications, respectively (Fig. 2A). In addition, the highest EO yield was recorded in moderate (FC75) and severe (FC50) water stress treatments treated with AA. The EO yield increased on HA, B and AA application by 35%, 29% and 57% compared with the control, respectively (Fig. 2B).

### Essential oil (EO) compositions

Based on the GC–MS and GC-Fid analysis, 15 compositions were identified in extracted EO, with the major compositions being carvacrol (38.87–43.32%) and gamma-terpinene (26.82–37.39%), alpha-terpinene (4.01–6.88%) and *p*-cymene (2.82–4.5%). The highest content of carvacrol and gamma-terpinene was obtained in FC75 and FC50 treated with AA. Also, the lowest content of the above-mentioned compositions was achieved in normal irrigation conditions without fertilization (control treatment). On average, the content of carvacrol and gamma-terpinene increased by 4%, 14% due to moderate (FC75) and 4%, 19% due to severe (FC50) water stress treatments. In addition, the application of AA enhanced carvacrol, gamma-terpinene, alpha-terpinene and *p*-cymene content by 6%, 19%, 46% and 18%, respectively (Table 5).

**Table 5:** Effect of irrigation regime and biofertilizer on essential oil compounds of savory (average of two years).

	Components	Treatments												
		RI	FC100 + C	FC100 + HA	FC100 + B	FC100 + AA	FC75 + C	FC75 + HA	FC75 + B	FC75 + AA	FC50 + C	FC50 + HA	FC50 + B	FC50 + AA
1	Alpha-thujene	929	1.21	2.01	2.21	2.17	1.67	1.96	2.37	2.16	1.95	2.04	2.11	1.68
2	Alpha-pinene	934	2.19	2.18	2.16	2. 2	2.14	1.17	0.26	0.31	1.3	1.15	1.21	0.9
3	Beta-Pinene	975	1.21	1.17	0.22	1.44	2.32	1.40	0.50	0.78	1.42	1.48	1.28	0.65
4	Beta-myrcene	990	1.45	2.12	2.42	2.14	1.83	2.01	2.26	1.89	1.97	2.07	1.90	1.82
5	Alpha-Phellandrene	1005	1.4	1.51	1.56	2.52	2.44	1.49	0.57	0.38	2.52	1.54	1.42	0.44
6	Alpha-terpinene	1017	**4.01**	**5.73**	**5.93**	**5.87**	**4.09**	**5.67**	**6.13**	**6.88**	**4.12**	**5.24**	**5.65**	**5.03**
7	*p*-cymene	1025	**2.82**	**3.62**	**3.93**	**3.35**	**3.08**	**3.33**	**4.5**	**2.98**	**3.16**	**3.53**	**3.80**	**4.32**
8	Limonene	1030	3.54	2.68	1.73	2.67	2.59	1.64	0.74	0.55	1.67	1.7	1.60	0.58
9	Gamma-terpinene	1060	**26.82**	**27.95**	**26.96**	**27.92**	**27.1**	**30.7**	**33.36**	**34.4**	**29.6**	**31.4**	**32.4**	**37.39**
10	Linalool	1099	1.12	1.10	1.14	1.11	2.10	1.12	0.19	0.11	1.10	0.14	0.12	0.18
11	Thymol	1292	2.10	0.17	1.16	1.13	1.10	1.17	0.13	0.19	1.11	0.14	0.17	0.18
12	Carvacrol	1305	**38.87**	**40.61**	**41.01**	**39.33**	**39.3**	**40.3**	**43.32**	**42.99**	**39.87**	**41.23**	**42.22**	**43.16**
13	Trans-caryophyllene	1425	2.18	1.13	1.42	0.30	1.15	1.23	0.12	0.60	1.20	0.46	0.75	0.19
14	Beta-bisabolene	1509	2.18	0.08	1.4	0.13	1.28	1.11	0.20	1.76	1.43	0.54	0.07	0.09
15	Spathulenol	1586	2.21	0.20	1.11	0.08	2.27	0.11	0.21	1.17	0.19	1.23	0.20	0.15
	Total identified (%)		93.31	92.26	94.36	92.36	94.46	94.41	94.86	97.15	92.61	93.89	94.9	96.76

Identification methods: MS, comparison of the mass spectrum with those of computer mass libraries Wiley, Adams, and NIST 08; RI, comparison of retention index with those reported in Adams and NIST 08; RI, linear retention indices on DB-5 MS column, experimentally determined using homolog series of *n*-alkanes Irrigation at 100% FC, 75% FC, and 50% FC, corresponding to full-irrigation, moderate water stress, and severe water stress, respectively, *C* control, *HA* humic acid, *B* bacterial, *AA* amino acid.

Main components are shown in bold.

### Chlorophyll concentration

The highest chlorophyll *a* (2.3 mg g^−1^ fresh weight) and *b* (1.2 mg g^−1^ fresh weight) concentrations were found under no-stress conditions (FC100) fertilized with AA. The lowest values (1.02 mg g^−1^ fresh weight for chlorophyll *a* and 0.6 mg g^−1^ fresh weight for chlorophyll *b*) were recorded under severe water stress (FC50) without fertilization treatment. Compared with FC100, chlorophyll *a* and *b* were reduced by 23% and 13% in moderate (FC75) and 32% and 29% in severe water stress (FC50), respectively. In addition, fertilization with HA, B and AA enhanced chlorophyll *a* concentration by 23%, 21% and 35.1% and chlorophyll *b* concentration by 23%, 17% and 33%, respectively (Fig. 2C,D).

### Carotenoid concentration

**Figure 3:**
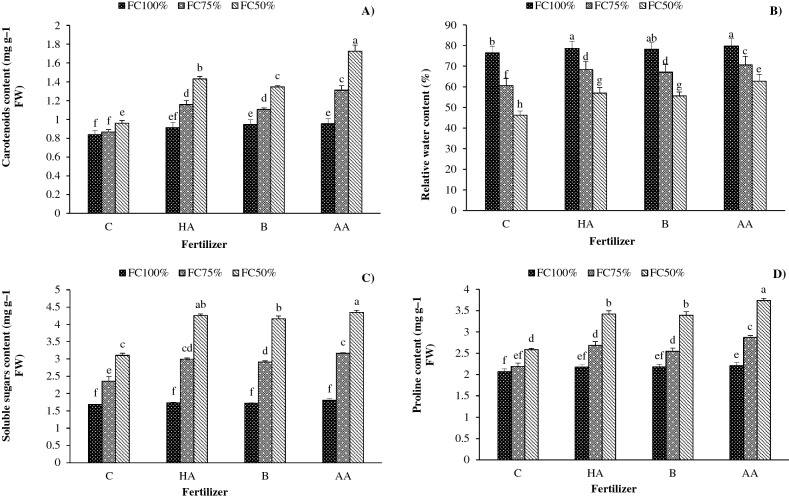
Interaction effects of irrigation regime and biofertilizer application on (**A**) carotenoids content, (**B**) relative water content, (**C**) soluble sugars content, and (**D**) proline content in savory. *C* control, *HA* humic acid, *B* bacterial, *AA* amino acid. Different lower-case letters above bars indicate significant differences at *P* < 0.05.

The carotenoid content was enhanced with increasing water stress levels. The highest carotenoid concentration (1.7 mg g^−1^ fresh weight) was measured in FC50 treated with AA. Compared with FC100, the carotenoid concentration was enhanced by 22 and 51% in FC75 and FC50 water stress. Also, fertilization with HA, B and AA enhanced carotenoid concentration by 31%, 27% and 49%, respectively, compared with the control (Fig. 3A).

### Relative water content (RWC)

The maximum (78%) and minimum (46%) RWC percentage values were related to normal irrigation conditions (FC100) treated with AA and severe water deficit (FC50) without fertilization, respectively. The RWC decreased with increasing water stress levels. Compared with FC100, RWC content was reduced by 15% and 29% in moderate (FC75) and severe water stress (FC50), respectively. Additionally, fertilization with HA, B and AA increased RWC by 11%, 10% and 16%, respectively, compared with the control (Fig. 3B).

### Soluble sugars concentration (SSC)

The maximum SSC (4.3 mg g^−1^ fresh weight) was obtained from FC50 fertilized with AA, followed by HA application (4.2 mg g^−1^ fresh weight). The SSC was enhanced with increasing water stress levels. Compared with FC100, the SSC increased by 64 and 128% in moderate (FC75) and severe water stress (FC50). Additionally, fertilization with HA, B and AA increased the SSC by 26%, 23% and 30%, respectively, compared with the control (Fig. 3C).

### Proline concentration

The fertilization with AA in FC50 strongly increased the proline concentration among different treatments. The proline concentration was enhanced with increasing water stress levels. Compared with FC100, proline concentration was enhanced by 19% and 52% in moderate (FC75) and severe water stress (FC50). Additionally, fertilization with HA, B and AA increased proline concentration by 21%, 18% and 29%, respectively, compared with the control (Fig. 3D).

### Enzymic activity

**Figure 4:**
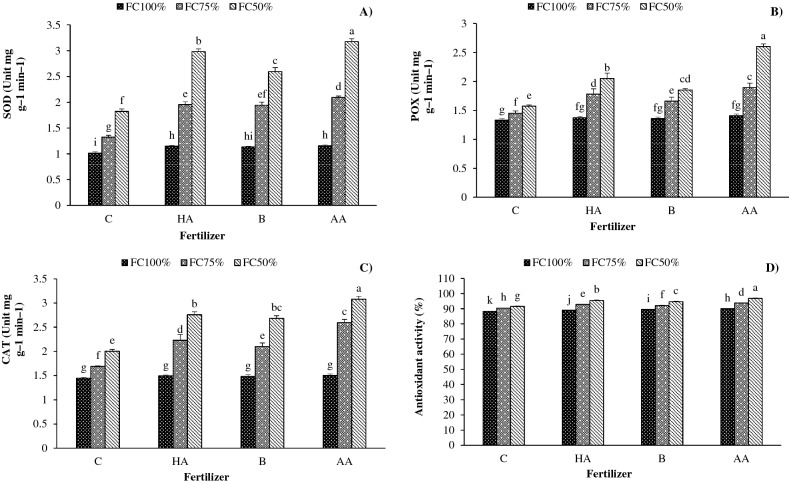
Interaction effects of irrigation regime and biofertilizer application on (**A**) superoxide dismutase activities, (**B**) peroxidase, (**C**) catalase, and (**D**) Antioxidant activity in savory. *C* control, *HA* humic acid, *B* bacterial, *AA* amino acid. Different lower-case letters above bars indicate significant differences at *P* < 0.05.

The maximum activity of SOD (3.2 unit mg^−1^ min^−1^), POX (2.6 unit mg^−1^ min^−1^) and CAT (3.08 unit mg^−1^ min^−1^) was recorded in severe water deficit (FC50) treated with AA. Increasing water stress levels enhanced the SOD, POX, and CAT activity. Compared with FC100, the activity of antioxidant enzymes was enhanced by 65%, 24 and 45.3% in FC75 and 137.8, 47.5 and 77.7% in FC50, respectively. Moreover, fertilization with HA, B and AA increased SOD activity by 46%, 36% and 54%, POX activity by 20%, 12% and 36% and CAT activity by 26%, 22% and 40%, respectively, when compared with no fertilization (Fig. 4A–C).

### Antioxidant activity

The antioxidant activity was enhanced with increasing water stress levels. The highest antioxidant activity (96.8%) was related to severe water stress (FC50) treated with AA. Compared with FC100, the antioxidant activity was enhanced by 3.5% and 6.1% in moderate and severe water stress. Also, fertilization with HA, B and AA enhanced the antioxidant activity by 2.5%, 2.2%, and 3.8%, respectively, compared with the control (Fig. 4D).

The correlation analysis revealed significant and positive correlations between dry matter yield and plant height (r = 0.98**), chlorophyll *b* (r = 0.97**), RWC (r = 0.96**), canopy diameter (r = 0.96**), lateral branch (r = 0.91**), chlorophyll *a* (r = 0.91**), soluble sugars content (r = 0.71*), and SOD (r = 0.60*) while essential oil content showed significant and positive correlations with carotenoid content (r = 0.99**), proline concentration (r = 0.99**), antioxidant activity (r = 0.98**), soluble sugars content (r = 0.95**), and enzymes activity including CAT (r = 0.98**), SOD (r = 0.98**), and POX (r = 0.96**) (Table 6).

**Table 6:** Correlation coefficient of morpho-physiological characteristics in summer savory (*Satureja hortensis* L.)

	PH	LB	DY	CD	EOC	EOY	Chla	Chlb	Carotenoid	RWC	Proline	SSC	CAT	SOD	POX
LB	0.941**														
DY	0.980**	0.911**													
CD	0.992**	0.919**	0.958**												
EOC	− 0.412	− 0.105	− 0.478	− 0.438											
EOY	0.490	0.740**	0.465	0.435	0.547										
Chla	0.968**	0.945**	0.910**	0.981**	− 0.286	0.531									
Chlb	0.981**	0.983**	0.967**	0.962**	− 0.265	0.635*	0.960**								
Carotenoid	− 0.288	0.031	− 0.361	− 0.312	0.984**	0.642*	− 0.156	− 0.136							
RWC	0.992**	0.905**	0.978**	0.992**	− 0.485	0.413	0.961**	0.960**	− 0.366						
Proline	− 0.492	− 0.197	− 0.569	− 0.507	0.988**	0.440	− 0.353	− 0.357	0.964**	− 0.562					
SSC	− 0.663*	− 0.390	− 0.706*	− 0.688*	0.948**	0.287	− 0.559	− 0.535	0.892**	− 0.724**	0.968**				
CAT	− 0.493	− 0.185	− 0.531	− 0.529	0.981**	0.499	− 0.395	− 0.340	0.956**	− 0.568	0.972**	0.967**			
SOD	− 0.544	− 0.249	− 0.602*	− 0.567	0.979**	0.411	− 0.426	− 0.408	0.949**	− 0.611*	0.986**	0.983**	0.978**		
POX	− 0.361	− 0.059	− 0.419	− 0.382	0.961**	0.577*	− 0.247	− 0.216	0.976**	− 0.434	0.940**	0.887**	0.945**	0.940**	
LB	− 0.493	− 0.184	− 0.535	− 0.530	0.979**	0.488	− 0.395	− 0.345	0.959**	− 0.570	0.974**	0.968**	0.989**	0.983**	0.948**

*PH* plant height, *LB* lateral branch, *DY* dry matter yield, *CD* Canopy diameter, *EOC* essential oil content, *EOY* essential oil yield, *Chla* chlorophyll a, *Chlb* chlorophyll b, *Carotenoid* carotenoids content, *RWC* relative water content, *Proline* proline content, *SSC* soluble sugars content, *CAT* catalase, *SOD* superoxide dismutase, *POX* peroxidase.

ns, * and **non-significant, significant at 5% and 1% probability level, respectively.

**Figure 5:**
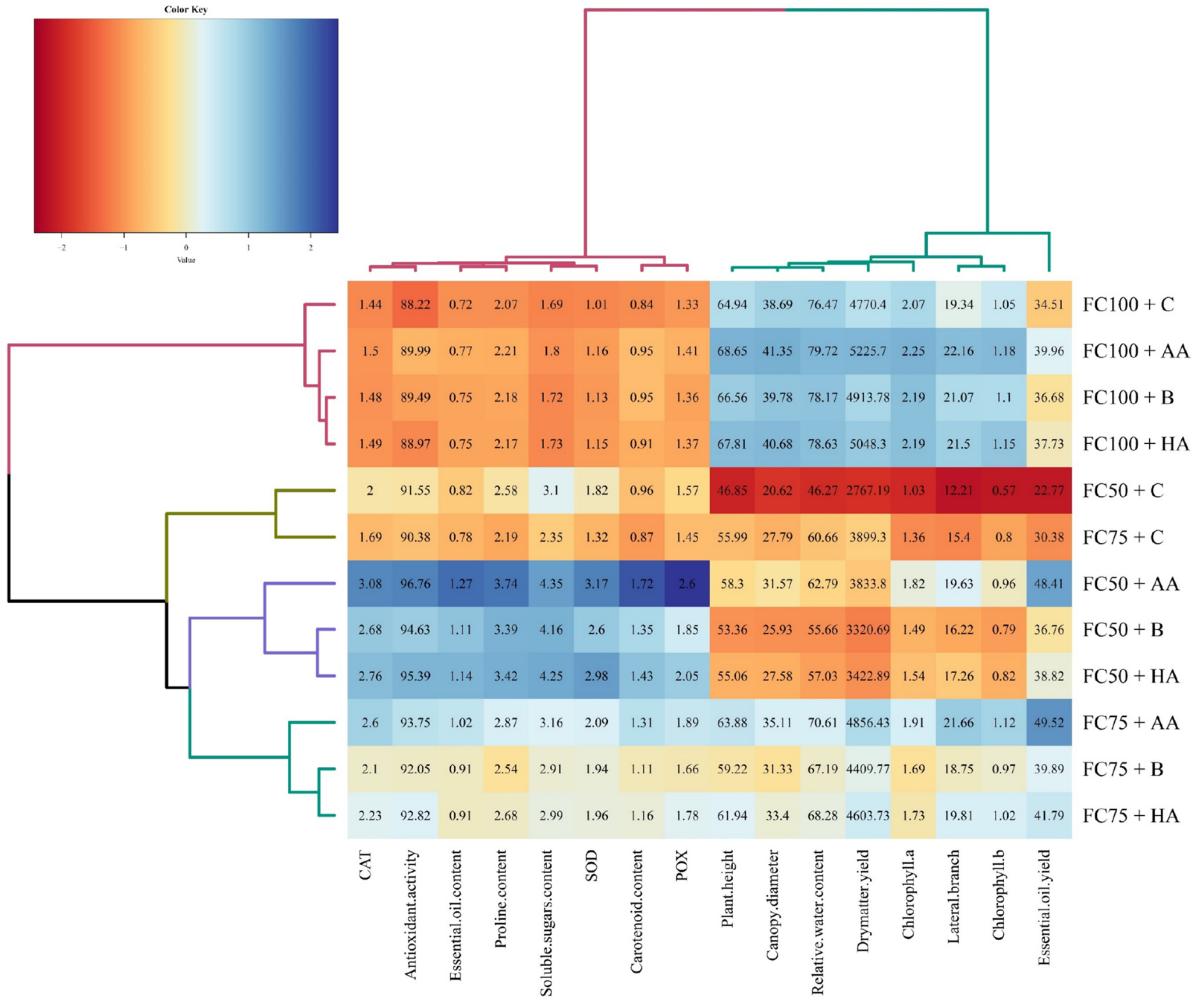
Heatmap clustering of irrigation regime (FC100, 100% field capacity; FC75, 75% field capacity; FC50, 50% field capacity) and fertilizer application (*C* control, *HA* humic acid, *B* bacterial, *AA* amino acid) sources based on morpho-physiological characteristics in summer savory (*Satureja hortensis* L.). The key color bar indicates standardized mean values (dark red indicates relatively low mean values; dark blue indicates relatively high mean values).

The heatmap clustering showed that the irrigation regimes significantly impacted the studied characteristics more than the biofertilizer treatments. All the biofertilizer treatments with the same irrigation regimes were classified into one group. The evaluated morpho-physiological variables clustered into two distinguished clusters: (1) essential oil yield, dry matter yield, plant height, canopy diameter, lateral branch, chlorophyll *a* and *b*, and relative water content; (2) carotenoid and soluble sugars contents, proline, POX, SOD, and CAT activities, antioxidant activity and essential oil content. All the classified variables in the first group decreased significantly with increasing water stress. In contrast, the classified variables in the second group significantly increased by increasing water stress (Fig. 5).

**Figure 6:**
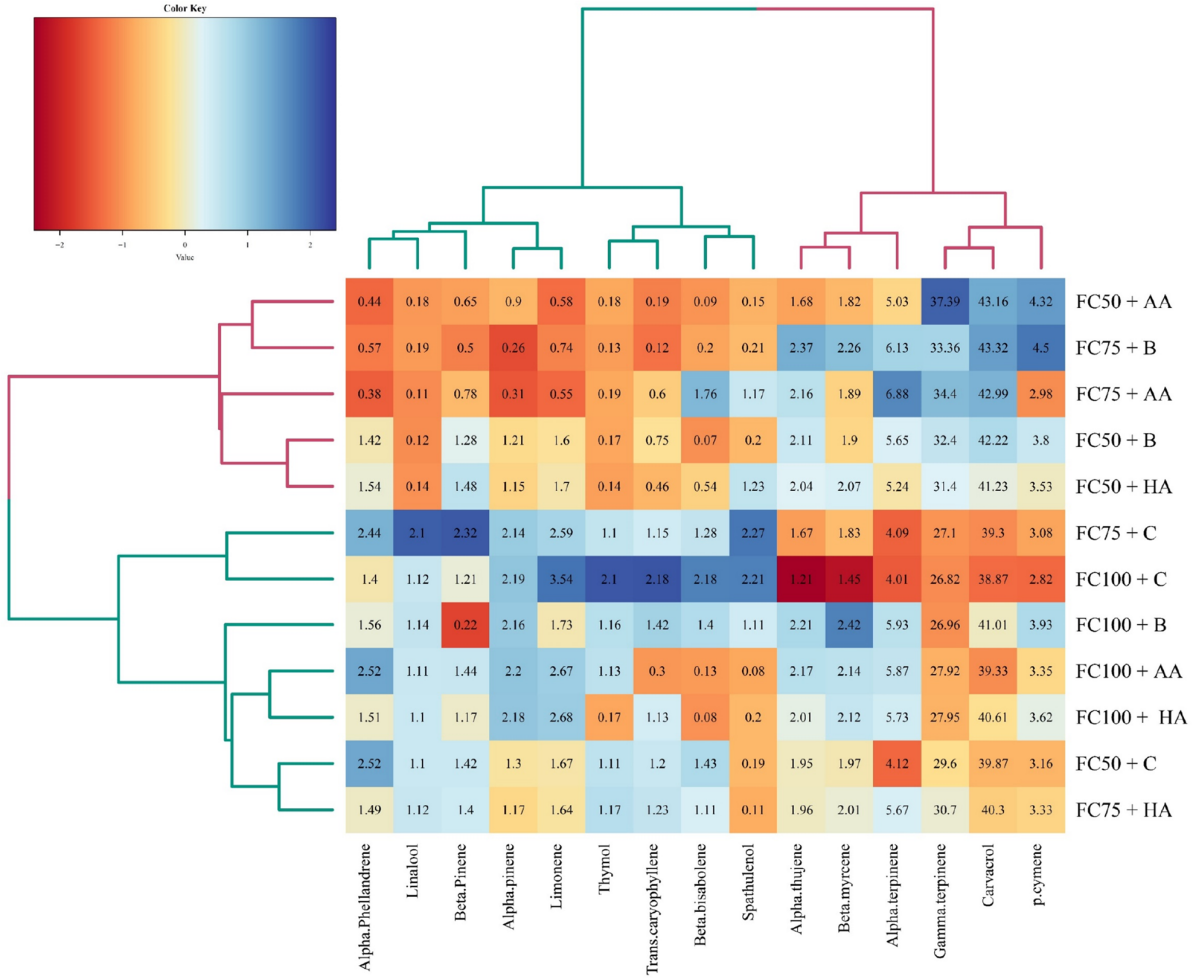
Heatmap clustering of irrigation regime (FC100, 100% field capacity; FC75, 75% field capacity; FC50, 50% field capacity) and fertilizer application (*C* control, *HA* humic acid, *B* bacterial, *AA* amino acid) sources based on essential oil constituents in in summer savory (*Satureja hortensis* L.). The key color bar indicates standardized mean values (dark red indicates relatively low mean values; dark blue indicates relatively high mean values).

The heatmap clustering of the essential oil compounds based on irrigation regimes and biofertilizers indicated higher amounts of alpha-phellandrene, linalool, beta-pinene, alpha-pinene, limonene, thymol, trans-caryophyllene, beta-bisabolene, and spathulenol were detected under normal-irrigation and control treatment of biofertilizer application. In contrast, these essential oil compounds were remarkably decreased under moderate water stress along with amino acid (FC75 + AA) and bacterial (FC75 + B) biofertilizers and severe water stress along with amino acid (FC50 + AA), bacterial (FC50 + B), and humic acid (FC50 + HA) biofertilizers (Fig. 6). In contrast, a higher amount of *p*-cymene, carvacrol, and gamma-terpinene were observed under moderate and severe water stress. The heatmap clustering shows moderate and severe water stress accompanied by amino acid and bacterial biofertilizers have the highest impact on essential oil metabolism (Fig. 6).

## Discussion

Our results show that plant height, lateral branches, canopy diameter, and dry matter yield all decreased due to moderate (FC75) and severe (FC50) water stress. Under water deficit stress conditions, nutrient absorption decreased around the root zone due to lower mobility and rate of mineral diffusion^36^. Water limitations affect cell differentiation, division, elongation and, finally, a reduction in photosynthesis rate and dry matter production^37^. Daszkowska-Golec et al.^38^ noted that water stress conditions reduce the entry of carbon dioxide into the closed stomata due to dehydration, reducing photosynthetic materials supply and plant productivity. Similarly, Amani Machiani et al.^39^ noted that thyme (*Thymus Vulgaris* L.) dry matter yield decreased due to moderate and severe drought stress by 27% and 40%, respectively. Also, under drought stress conditions, AA application positively affected the plant yield components and dry matter yield and also decreased the negative impacts of drought stress^40^. The proposed mechanism for AA improving plant productivity is by improving nutrient use efficiency and increasing photosynthetic rates, improving growth^16,41^.

The results showed moderate and severe water stress conditions increased the EO content, yield, and major constituents, including carvacrol and gamma-terpinene. The production of secondary metabolites such as EOs, alkaloids and flavonoids in medicinal and aromatic plants is considered one of the defense mechanisms to increase plant resistance under stressful conditions^42^. Under drought stress, the massive supply of NADPH + H^+^ in plant cells adversely affects the photosynthesis cycle. Therefore, EOs compounds' productivity using NADPH + H + stored in plant cells balances the NADP+/NADPH+ H+ ratio and improves the photosynthesis cycle^43^. Among different fertilizer treatments, foliar application of AA enormously improved EO quantity and quality by increasing the main EO constituents, including carvacrol, gamma-terpinene, alpha-terpinene and *p*-cymene. Most EO constituents, such as terpenes composed of an isoprene structure. The isoprene productivity depends on nutrient availability and biomolecules such as ATP, NADPH, and acetyl-CoA^44^. The application of AA enhanced essential oil productivity and major EO constituents by increasing the availability of intermediate compounds, including ATP and NADPH. Similarly, Talaat et al.^45^ noted that applying amino acid bioregulators improves the essential oil quantity and quality of Khella (*Ammi visnaga* L.).

In the present study, seedlings' chlorophyll content (a and b) decreased under moderate and severe water stress conditions. The reduction in the photosynthesis pigments under water deficit stress conditions may be caused by chloroplast breakdown and inhibition of its biosynthesis by enhancing the synthesis of reactive oxygen species (ROS), degradation of chlorophyll precursor, and up-regulation chlorophyllase activity^40^. These conditions increase lipid peroxidation, leading to chlorophyll and photosynthesis pigment degradation^46^. Similar results were found by Rezaei-Chiyaneh et al.^1^ study, who noted that the chlorophyll concentration in black cumin (*Nigella sativa* L.) significantly decreased under drought stress conditions. In contrast, carotenoid content was increased under drought stress conditions due to the higher susceptibility of these compounds to oxidative stress. Therefore, an increase in carotenoid content, as a non-enzymatic antioxidant, under water deficit stress conditions plays a vital role in improving plant tolerance and decreasing the detrimental effects of oxidative stress^47,48^. In addition, chlorophyll (*a* and *b*) and carotenoid content increased because of AA foliar application. AA, as an antistress compound, enhances the activity of antioxidant enzymes and proline concentration, leading to decreased ROS harmful effects in the degradation of chlorophylls and carotenoid membranes. Noroozlo et al.^49^ stated that the higher chlorophyll and carotenoid content in the lettuce plants could be due to the reduction in chlorophyll degradation and the stimulating impacts of AAs on chlorophyll and carotenoid biosynthesis.

The results showed that the maximum proline content was achieved from severe water stress (FC50) treatment with AA foliar application. Under drought-stress conditions, plants can alter water relations to maintain cellular functions. For example, plants exhibit osmotic adjustment by synthesizing and accumulating compatible solutes such as free amino acids, sugars, and proline. Also, Hanif et al.^50^ noted that proline-based amino acid foliar application and increased proline content would increase osmoprotectants' productivity. Therefore, enhancing proline content through foliar application decreased their negative impacts by stabilizing the membranes, maintaining the osmotic balance and cell turgor, preventing electrolyte leakage, and maintaining the ROS levels in normal ranges (detoxification of ROS)^51^.

RWC is commonly used to quantify plant water status in terms of biochemical and physiological consequences of water stress in plant cells^52^. The results revealed that RWC was decreased under moderate and severe water stress conditions due to increased water loss from savory leaves through transpiration and decreasing water uptake from roots. In this situation, RWC decreased due to reduced leaf water potential^47^. In addition, the AA application strongly increased RWC content in the seedlings. Under drought stress conditions, reducing osmotic pressure by increasing osmotic adjustment such as SSC and proline in the cells helps maintain leaves’ turgor pressure. The application of AA enhanced RWC content under stressful conditions through increasing osmotic adjustment and membrane integrity^53^.

CAT, SOD and POX activity were enhanced under severe water stress treated with AA. Under water stress conditions, the antioxidant enzyme activity plays a vital role in eliminating ROS compounds such as superoxide and hydrogen peroxide (H_2_O_2_). The higher antioxidant enzyme activity reduces ROS compounds in the cells, reducing membrane damage and balancing the performance of cellular activities such as photosynthesis^54^. Also, increased antioxidant activity due to AA foliar application could be attributed to higher nitrogen metabolizing enzyme activity because of amino acid availability^16^. In a study, Shafiq et al.^17^ reported that a higher proline concentration under drought stress conditions protects macromolecules and enzymes from oxidative damage and increases the activities of the enzymes. Therefore, it can be concluded that AA foliar application can enhance plant protection against drought stress by enhancing organic osmolytes such as proline, SSC, and antioxidant enzyme activity.

## Conclusion

Our study revealed that water stress negatively affects savory seedlings, reducing dry matter production by 11% and 33% under moderate and severe water deficit stress. However, foliar application of amino acid enhanced dry matter yield, essential oil content and savory yield by 22%, 31% and 57%, compared with the treatment with no fertilization. Foliar application of amino acid improved savory essential oil quality by increasing essential oil components, including carvacrol, gamma-terpinene, alpha-terpinene and *p*-cymene. We conclude that the foliar application of amino acid could effectively increase plant tolerance against water deficit stress while improving the essential oil quantity and quality of medicinal and aromatic plants.

## Data Availability

The authors confirm that the data supporting the findings of this study are available within the article.

## References

[1] Rezaei-Chiyaneh E, Seyyedi SM, Ebrahimian E, Moghaddam SS, Damalas CA (2018). Exogenous application of gamma-aminobutyric acid (GABA) alleviates the effect of water deficit stress in black cumin (*Nigella sativa* L.). Ind. Crops Prod..

[2] Biglari T, Maleksaeidi H, Eskandari F, Jalali M (2019). Livestock insurance as a mechanism for household resilience of livestock herders to climate change: Evidence from Iran. Land Use Policy.

[3] Harrison MT, Evans JR, Moore AD (2012). Using a mathematical framework to examine physiological changes in winter wheat after livestock grazing. 1. Model derivation and coefficient calibration. Field Crops Res..

[4] Harrison MT, Evans JR, Moore AD (2012). Using a mathematical framework to examine physiological changes in winter wheat after livestock grazing. 2. Model validation and effects of grazing management. Field Crops Res..

[5] Thomason W, Battaglia M (2020). Early defoliation effects on corn plant stands and grain yield. Agron. J..

[6] Raza MA, Gul H, Wang J, Shehryar Yasin H, Qin R, Bin Khalid MH, Naeem M, Feng LY, Iqbal N, Gitari H, Ahmad S, Battaglia M, Ansar M, Yang F, Yang WJ (2021). Land productivity and water use efficiency of maize-soybean strip intercropping systems in semi-arid areas: A case study in Punjab Province, Pakistan. J. Clean. Prod..

[7] Roy R, Núñez-Delgado A, Sultana S, Wang J, Munir A, Battaglia ML, Sarker T, Seleiman MF, Barmon M, Zhang R (2021). Additions of optimum water, spent mushroom compost and wood biochar to improve the growth performance of *Althaea rosea* in drought-prone coal-mined spoils. J. Environ. Manag..

[8] Seleiman MF, Al-Suhaibani N, Ali N, Akmal M, Alotaibi M, Refay Y, Dindaroglu T, Abdul-Wajid HH, Battaglia ML (2021). Drought stress impacts on plants and different approaches to alleviate its adverse effects. Plants.

[9] Bell LW, Harrison MT, Kirkegaard JA (2015). Dual-purpose cropping-capitalising on potential grain crop grazing to enhance mixed-farming profitability. Crop Pasture Sci..

[10] Ibrahim A, Harrison M, Meinke H, Fan Y, Johnson P, Zhou M (2018). A regulator of early flowering in barley (*Hordeum vulgare* L.). PLoS ONE.

[11] Liu K, Harrison MT, Hunt J, Angessa TT, Meinke H, Li C (2020). Identifying optimal sowing and flowering periods for barley in Australia: A modelling approach. Agric. Meteorol..

[12] Sharma P, Jha AB, Dubey RS, Pessarakli M (2012). Reactive oxygen species, oxidative damage, and antioxidative defense mechanism in plants under stressful conditions. J. Bot..

[13] Seleiman MF, Aslam MT, Alhammad BA, Hassan MU, Maqbool R, Chattha MU, Khan I, Gitari HI, Uslu OS, Roy R, Battaglia ML (2022). Salinity stress in wheat: Effects, mechanisms and management strategies. Phyton.

[14] Rogers H, Munné-Bosch S (2016). Production and scavenging of reactive oxygen species and redox signaling during leaf and flower senescence: Similar but different. Plant Physiol..

[15] Wahab A, Abdi G, Saleem MH, Ali B, Ullah S, Shah W, Mumtaz S, Yasin G, Muresan CC, Marc RA (2022). Plants’ physio-biochemical and phyto-hormonal responses to alleviate the adverse effects of drought stress: A comprehensive review. Plants.

[16] García-García AL, García-Machado FJ, Borges AA, Morales-Sierra S, Boto A, Jiménez-Arias D (2020). Pure organic active compounds against abiotic stress: A biostimulant overview. Front. Plant Sci..

[17] Shafiq S, Akram NA, Ashraf M, García-Caparrós P, Ali OM, Abdel Latef AAH (2021). Influence of glycine betaine (Natural and synthetic) on growth, metabolism and yield production of drought-stressed maize (*Zea mays* L.) plants. Plants.

[18] Anli M, Baslam M, Tahiri A, Raklami A, Symanczik S, Boutasknit A, Ait-El-Mokhtar M, Ben-Laouane R, Toubali S, Ait Rahou Y, Ait Chitt M, Oufdou K, Mitsui T, Hafidi M, Meddich A (2020). Biofertilizers as strategies to improve photosynthetic apparatus, growth, and drought stress tolerance in the date palm. Front. Plant Sci..

[19] Singh D, Thapa S, Geat N, Mehriya ML, Rajawat MVS, Rakshit A, Meena VS, Parihar M, Singh HB, Singh AK (2021). Biofertilizers: Mechanisms and application biofertilizers. Biofertilizers.

[20] Fotohi Chiyaneh S, Rezaei-Chiyaneh E, Amirnia R, Keshavarz Afshar R, Siddique KHM (2022). Changes in the essential oil, fixed oil constituents, and phenolic compounds of ajowan and fenugreek in intercropping with pea affected by fertilizer sources. Ind. Crops Prod..

[21] Fathi R, Mohebodini M, Chamani E, Sabaghnia N (2021). Morphological and phytochemical variability of *Satureja hortensis* L. accessions: An effective opportunity for industrial production. Ind. Crops Prod..

[22] Alizadeh A, Moghaddam M, Asgharzade A, Sourestani MM (2020). Phytochemical and physiological response of *Satureja hortensis* L. to different irrigation regimes and chitosan application. Ind. Crops Prod..

[23] Ho CKM, Jackson T, Harrison MT, Eckard RJ (2014). Increasing ewe genetic fecundity improves whole-farm production and reduces greenhouse gas emissions intensities: 2. Economic performance. Anim. Prod. Sci..

[24] Harrison MT, Cullen BR, Mayberry DE, Cowie AL, Bilotto F, Badgery WB (2021). Carbon myopia: The urgent need for integrated social, economic and environmental action in the livestock sector. Glob. Change Biol..

[25] Zhang H, Li Y, Zhu JK (2018). Developing naturally stress-resistant crops for a sustainable agriculture. Nat. Plants.

[26] Yan H, Harrison MT, Liu K, Wang B, Feng P, Fahad S, Meinke H, Yang R, Liu DL, Archontoulis S, Huber I, Tian X, Man J, Zhang Y, Zhou M (2022). Crop traits enabling yield gains under more frequent extreme climatic events. Sci. Total Environ..

[27] Namazi Y, Rezaei-Chiyaneh E, Siavash Moghaddam S, Leonardo Battaglia M (2022). The effects of microbial inoculation and intercropping on yield and active ingredients of savory (*Satureja hortensis* L.) intercropped with common bean (*Phaseolus vulgaris* L.). Int. J. Environ. Sci. Technol..

[28] Levitt J (1980). Responses of Plant to Environmental Stress: Water, Radiation, Salt and Other Stresses.

[29] Lichtenthaler HK (1987). Chlorophylls and carotenoids: Pigments of photosynthetic biomembranes. Methods Enzymol..

[30] Bates LS, Waldren RP, Teare ID (1973). Rapid determination of free proline for water-stress studies. Plant Soil.

[31] Irigoyen JJ, Emerich DW, Sanchez-Diaz M (1992). Water stress induced changes in concentrations of proline and total soluble sugars in nodulated alfalfa (*Medicago sativa*) plants. Physiol. Plant..

[32] Dhindsa RS, Plumb-dhindsa P, Thorpe TA (1981). Leaf senescence: Correlated with increased levels of membrane permeability and lipid peroxidation, and decreased levels of superoxide dismutase and catalase. J. Exp. Bot..

[33] Kar M, Feierabend J (1984). Metabolism of activated oxygen in detached wheat and rye leaves and its relevance to the initiation of senescence. Planta.

[34] Aebi H, Bergmeyer HU (1974). Catalases. Methods of Enzymatic Analysis.

[35] Brand-Williams W, Cuvelier ME, Berset C (1995). Use of a free radical method to evaluate antioxidant activity. LWT Food Sci. Technol..

[36] Hussain HA, Hussain S, Khaliq A, Ashraf U, Anjum SA, Men S, Wang L (2018). Chilling and drought stresses in crop plants: Implications, cross talk, and potential management opportunities. Front. Plant Sci..

[37] Manavalan LP, Guttikonda SK, Phan Tran LS, Nguyen HT (2009). Physiological and molecular approaches to improve drought resistance in soybean. Plant Cell Physiol..

[38] Daszkowska-Golec A, Szarejko I (2013). Open or close the gate—Stomata action under the control of phytohormones in drought stress conditions. Front. Plant Sci..

[39] Amani Machiani M, Javanmard A, Morshedloo MR, Aghaee A, Maggi F (2021). *Funneliformis mosseae* inoculation under water deficit stress improves the yield and phytochemical characteristics of thyme in intercropping with soybean. Sci. Rep..

[40] Rezaei-Chiyaneh E, Mahdavikia H, Hadi H, Alipour H, Kulak M, Caruso G, Siddique KHM (2021). The effect of exogenously applied plant growth regulators and zinc on some physiological characteristics and essential oil constituents of moldavian balm (*Dracocephalum moldavica* L.) under water stress. Physiol. Mol. Biol. Plants.

[41] Li J, Van Gerrewey T, Geelen D (2022). A meta-analysis of biostimulant yield effectiveness in field trials. Front. Plant Sci..

[42] Faridvand S, Rezaei-Chiyaneh E, Battaglia ML, Gitari HI, Raza MA, Siddique KHM (2022). Application of bio and chemical fertilizers improves yield, and essential oil quantity and quality of moldavian balm (*Dracocephalum moldavica* L.) intercropped with mung bean (*Vigna radiata* L.). Food Energy Secur..

[43] Kleinwächter M, Selmar D (2015). New insights explain that drought stress enhances the quality of spice and medicinal plants: Potential applications. Agron. Sustain. Dev..

[44] Ormeno E, Fernandez C (2012). Effect of soil nutrient on production and diversity of volatile terpenoids from plants. Curr. Bioact. Compd..

[45] Talaat IM, Khattab HI, Ahmed AM (2014). Changes in growth, hormones levels and essential oil content of *Ammi visnaga* L. plants treated with some bioregulators. Saudi J. Biol. Sci..

[46] Abd Elbar OH, Farag RE, Shehata SA (2019). Effect of putrescine application on some growth, biochemical and anatomical characteristics of *Thymus vulgaris* L. under drought stress. Ann. Agric. Sci..

[47] Farooq MA, Niazi AK, Akhtar J, Saifullah, Farooq M, Souri Z, Karimi N, Rengel Z (2019). Acquiring control: The evolution of ROS-induced oxidative stress and redox signaling pathways in plant stress responses. Plant Physiol. Biochem..

[48] Mohammadi H, Amirikia F, Ghorbanpour M, Fatehi F, Hashempour H (2019). Salicylic acid induced changes in physiological traits and essential oil constituents in different ecotypes of *Thymus kotschyanus* and *Thymus vulgaris* under well-watered and water stress conditions. Ind. Crops Prod..

[49] Noroozlo YA, Souri MK, Delshad M (2019). Stimulation effects of foliar applied glycine and glutamine amino acids on lettuce growth. Open Agric..

[50] Hanif S, Saleem MF, Sarwar M, Irshad M, Shakoor A, Wahid MA, Khan HZ (2021). Biochemically triggered heat and drought stress tolerance in rice by proline application. J. Plant Growth Regul..

[51] Hayat S, Hayat Q, Alyemeni MN, Wani AS, Pichtel J, Ahmad A (2012). Role of proline under changing environments: A review. Plant Signal. Behav..

[52] Amani Machiani M, Javanmard A, Morshedloo MR, Janmohammadi M, Maggi F (2021). *Funneliformis mosseae* application improves the oil quantity and quality and eco-physiological characteristics of soybean (*Glycine max* L.) under water stress conditions. J. Soil Sci. Plant Nutr..

[53] Hammad SAR, Ali OAM (2014). Physiological and biochemical studies on drought tolerance of wheat plants by application of amino acids and yeast extract. Ann. Agric. Sci..

[54] Dong S, Jiang Y, Dong Y, Wang L, Wang W, Ma Z, Yan C, Ma C, Liu L (2019). A study on soybean responses to drought stress and rehydration. Saudi J. Biol. Sci..

